# Application of a Bayesian dominance model improves power in quantitative trait genome-wide association analysis

**DOI:** 10.1186/s12711-017-0284-7

**Published:** 2017-01-14

**Authors:** Jörn Bennewitz, Christian Edel, Ruedi Fries, Theo H. E. Meuwissen, Robin Wellmann

**Affiliations:** 1 Institute of Animal Science, University of Hohenheim, 70593 Stuttgart, Germany; 2 Institute of Animal Breeding, Bavarian State Research Center for Agriculture, 85580 Grub, Germany; 3 Chair of Animal Breeding, Technical University of Munich, Liesel-Beckmann-Strasse 1, 85354 Freising, Germany; 4 Institute of Animal and Aquacultural Science, Norwegian University of Life Science, 1450 Aas, Norway

## Abstract

**Background:**

Multi-marker methods, which fit all markers simultaneously, were originally tailored for genomic selection purposes, but have proven to be useful also in association analyses, especially the so-called BayesC Bayesian methods. In a recent study, BayesD extended BayesC towards accounting for dominance effects and improved prediction accuracy and persistence in genomic selection. The current study investigated the power and precision of BayesC and BayesD in genome-wide association studies by means of stochastic simulations and applied these methods to a dairy cattle dataset.

**Methods:**

The simulation protocol was designed to mimic the genetic architecture of quantitative traits as realistically as possible. Special emphasis was put on the joint distribution of the additive and dominance effects of causative mutations. Additive marker effects were estimated by BayesC and additive and dominance effects by BayesD. The dependencies between additive and dominance effects were modelled in BayesD by choosing appropriate priors. A sliding-window approach was used. For each window, the R. Fernando window posterior probability of association was calculated and this was used for inference purpose. The power to map segregating causal effects and the mapping precision were assessed for various marker densities up to full sequence information and various window sizes.

**Results:**

Power to map a QTL increased with higher marker densities and larger window sizes. This held true for both methods. Method BayesD had improved power compared to BayesC. The increase in power was between −2 and 8% for causative genes that explained more than 2.5% of the genetic variance. In addition, inspection of the estimates of genomic window dominance variance allowed for inference about the magnitude of dominance at significant associations, which remains hidden in BayesC analysis. Mapping precision was not substantially improved by BayesD.

**Conclusions:**

BayesD improved power, but precision only slightly. Application of BayesD needs large datasets with genotypes and own performance records as phenotypes. Given the current efforts to establish cow reference populations in dairy cattle genomic selection schemes, such datasets are expected to be soon available, which will enable the application of BayesD for association mapping and genomic prediction purposes.

## Background

With the advent of dense single nucleotide polymorphisms (SNP) panels, it has become possible to exploit linkage disequilibrium (LD) between SNPs and genes that are involved in complex or quantitative trait variation, with the aim to map genes that underlie trait variation and to predict genomic values [1]. Genome-wide association studies (GWAS) scan the genome systematically to identify SNPs that are significantly associated with the trait of interest. Various methods to conduct GWAS are available. Single-SNP analyses are widely used, where one SNP is tested at a time for significance. The SNP genotype is usually treated as a fixed effect in a mixed linear model. Correction for the effects of population structure is done by fitting simultaneously a random polygenic term in the model [2]. For each SNP, a test statistic and an error probability for the trait association is obtained in a ‘frequentist’ manner, which can conveniently be used for post-GWAS analyses, such as false discovery rate calculations [3], or for meta-analyses, e.g. [4]. However, with dense SNP panels, the level of multiple-testing can be enormous, which needs a very stringent significance threshold in order to prevent an inflation of type one errors. In addition, the effect of a gene can be captured only in part by a single marker due to imperfect LD, but might be better explained by using jointly the SNPs that surround the gene. To overcome these problems, multi-marker methods have been proposed, which fit all SNPs simultaneously as random effects in the model [5]. These models were originally tailored for genomic prediction or selection purposes [6], but have proven to be useful also in association analyses [7]. The simulation study of Sahana et al. [8] revealed that Bayesian multi-marker association analysis has a higher power than single-SNP analysis. These authors used also a window-based approach, where consecutive SNPs within 1 cM were used to build a window. Inference was drawn for each window by considering these window SNPs jointly. Legarra et al. [9] compared linkage and linkage disequilibrium analysis (known as LDLA), single-marker mixed model association analysis, and Bayesian whole-genome association analysis using a real data structure. They did not report a clear superiority of one method, but recommended to apply more than one method to real data.

In Bayesian analysis, inference on unknowns is drawn from their posterior distributions. Recently, Fernando and Garrick [5] and Fernando et al. [10] developed a method to control false positive results in multi-marker Bayes GWAS, which can be straightforwardly implemented in MCMC-based algorithms. This method controls the proportion of false positives by calculating the posterior probability of association of a trait with each SNP or each window of consecutive SNPs.

To the best of our knowledge, the Bayesian models mentioned above consider only additive gene effects. However, dominance is a non-negligible source of complex trait variation [11–13]. Bolormaa et al. [14] used a large-scale experiment with about 10,000 bovine individuals, which were phenotyped for 16 traits and genotyped with dense SNP panels. They conducted a GWAS using single-marker regression analysis and found many trait-associated SNPs that had a dominance effect. Moreover, the estimated dominance variance across the traits was between 0 and 42% of the phenotypic variance, with a median of 5%. It is well known that additive and dominance effects are dependent in a complicated manner [15], as described in the next section.

Verbyla et al. [16, 17] proposed a Bayesian stochastic search variable selection method, which was named BayesC by these authors. In a recent study, we extended this BayesC method by accounting for dominance, resulting in the BayesD method, with the three sub-models BayesD1, 2 and 3 [18]. These sub-models differ in the way dependencies between additive and dominance effects are modelled. Simulation studies showed that these models increased the accuracy of predicted genetic values by about 15%. Moreover, application on a real dairy cattle dataset revealed that the use of these BayesD models increased the prediction accuracy of cow’s yield deviations for milk fat yield compared to a G-BLUP analysis without dominance [19]. Hence, it seems worthwhile to investigate the use of BayesD models in association analyses also. Therefore, the aim of our study was to compare the power and precision of BayesD and BayesC in a GWAS. The analysis was conducted on simulated datasets, using a protocol that simulated many segregating genes and accounted for the dependencies between additive and dominant gene effects. The models were also applied to a real dairy cattle dataset.

## Methods

### Simulation protocol

A forward simulation approach was used to generate a Fisher-Wright diploid population with a genome that consisted of one chromosome that was 1 Morgan (M) long. The mutation rate was 10^−8^/bp/meiosis. In total, 1051 generations were generated. For the first 650 generations, the effective population size was Ne = 600, and then decreased to 100 in the following 350 generations, with a fast decline in the last generations. This decline was chosen in order to create an LD pattern as observed in bovine populations [20]. From generation 1000 to 1051, the Ne remained constant at 100. In the last generation, the sample size was N = 1500. According to the scaling by Ne and genome length argument given in [21], a simulated population with an N of 1500 and an Ne of 100 corresponds to a population of 45,000 individuals with a genome of 30 M, with an Ne of 100, or of 450,000 individuals with an Ne of 1000. In total, ten populations were simulated. The average number of segregating SNPs (minor allele frequency (MAF) higher than 0.01) in the last generation was about 7k. From these 7k SNPs, 2k, 1k and 0.5k SNPs were chosen based on a MAF higher than 0.03 and equal distances between SNPs. This corresponds to a marker density of 20Ne, 10Ne and 5Ne per Morgan. Scaling this to a population of individuals with a genome of 30 M and an Ne of 1000, it reflects marker densities of 600k, 300k, and 150k, respectively. If the Ne is equal to 100, this results in marker densities of 60k, 30k, and 15k, respectively. Note that these scaling arguments were derived for genomic predictions [21]. In our study, it was assumed that these scaling arguments also approximately hold for GWAS using genomic prediction models.

**Fig. 1:**
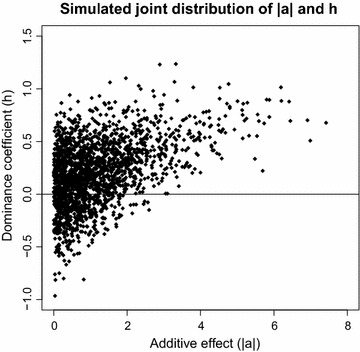
Scatterplot of the simulated joint distribution of the absolute value of additive effects ($\left| a \right|$) and dominance coefficients ($h = d/\left| a \right|)$

For each population, five traits were simulated as follows. In the last generation, 15 of the 7k SNPs with a MAF higher than 0.05 were randomly selected as causal mutations. The minimum distance between two quantitative trait loci (QTL) was 2 cM. The additive effect ($a$) represents half the difference between the alternative homozygous genotypes and the dominance effect ($d$) represents the deviation of the heterozygous genotype from the mean of the two alternative homozygous genotypes. The distribution of the dominance coefficient $h = d/\left| a \right|$ was assumed to be $h\,\sim\,N\left( {0.2,0.3^{2} } \right)$ [22]. The distribution of $a$ was $a|h\,\sim\,N\left( {0,\exp \left( {3h} \right)} \right)$ [15]. A scatterplot of these distributions is in Fig. 1, which shows that alleles with small additive effects had high variable dominance effects. As the size of the additive effect increased, the dominance coefficient was likely to become more positive and, on average, increased in size. Hence, on average, the genetic value of heterozygous genotypes was above the mean of the two alternative homozygous genotypes. Moreover, overdominance was a rare event. This pattern reflects the results of Caballero and Keightley [23], who found that genes with large additive effects likely have larger dominance coefficients. It is also in agreement with the well-known Kacser–Burns model [24], and with theoretical derivations about the contribution of dominance to the variation of quantitative traits [15].

From these simulated genotype values, the breeding values, dominance deviations, and genetic values of the individuals were calculated, following the derivations in Falconer and Mackay [25]. For an individual with genotype $x$ ($x$ representing the number of copies of the mutant allele at the causative mutation, $x$ = 0, 1, or 2), the breeding value ($BV$) is:

1 $$ BV\left( x \right) = \mathop \sum \limits_{j = 1}^{Q} \left( {x_{j} - 2p_{j} } \right)\alpha_{j} , $$

where $$ \alpha_{j} = a_{j} + \left( {q_{j} - p_{j} } \right)d_{j} $$ is the substitution effect, $$ p_{j} $$ the frequency of the mutant allele, $$ q_{j} = 1 - p_{j} , $$ and $$ Q $$ is the number of simulated causative mutations (i.e. 15). The dominance deviation ($$ DV $$) is:

2 $$ DV\left( x \right) = \mathop \sum \limits_{j = 1}^{Q} - d_{j} x_{j} \left( {x_{j} - 1 - 2p_{j} } \right) - 2p_{j}^{2} d_{j} . $$

The genetic value ($GV$) is:

3 $$ GV\left( x \right) = \mathop \sum \limits_{j = 1}^{Q} \left( {a_{j} + \left( {2 - x_{j} } \right)d_{j} } \right)x_{j} . $$

The additive genetic variance was calculated as the variance of the $BV$ and the dominance variance as the variance of the $DV$. The residuals were sampled from a normal distribution with mean zero and a residual variance chosen such that the narrow sense heritability was equal to 0.3 for each trait. The mean (median) of the dominance variance as the proportion of the phenotypic variance was equal to 0.1 (0.08), but varied between 0.01 and 0.29 across the simulated traits. The expected inbreeding depression was calculated as $\sum\nolimits_{j = 1}^{Q} {2p_{j} q_{j} d_{j} }$ and was on average 0.023 times the phenotypic standard deviation across all simulated traits.

We simulated 50 replicates. For each replicate, we chose four marker densities, i.e. 7k, 2k, 1k, and 0.5k. The causal mutations were removed, except for the 7k dataset. For this dataset, no selection on SNPs was conducted (except for MAF), and hence, it mimics a situation where the full sequence is known and the causative mutations are among the full set of SNPs.

### Bayesian models

The three BayesD sub-models of Wellmann and Bennewitz [18] differ in the way the complicated relationships between additive and dominance effects are modelled. Sub-model BayesD3 performed best in their simulation study, followed by BayesD2. However, BayesD2 showed better mixing properties in the MCMC analysis. Since the number of iterations is an issue in the application of these models, we used BayesD2 in this study, which will be thereafter named simply BayesD. BayesD1 was used in some preliminary analyses, in which it was slightly less powerful than BayesD2 (Wellmann and Bennewitz, unpublished results). Therefore, it was not included in this study.

A full description of BayesD can be found in [18]. Only essential issues will be described here. The following general linear regression model was applied for BayesD:

$$ {\mathbf{y}} = 1_{{\mathbf{n}}} \mu + {\mathbf{X}}{\tilde{\mathbf{a}}} + {\mathbf{W}}{\tilde{\mathbf{d}}} + {\mathbf{E}}, $$

where ${\mathbf{y}}$ is the vector of the observations of the ${\text{n}}$ individuals, ${\mathbf{1}}_{{\mathbf{n}}}$ is a vector of ${\text{n}}$ ones and $\mu$ is the general mean. Vector ${\tilde{\mathbf{a}}}$ contains the random additive and vector ${\tilde{\mathbf{d}}}$ the random dominance effects of the $M$ SNPs. The SNP genotypes are coded as ‘0 0’, ‘0 1’, and ‘1 1’. ${\mathbf{X}}$ is a known $N \times M$ matrix and contains the number of copies of 1-alleles at the SNPs for each individual, i.e. the gene content. ${\mathbf{W}}$ is a known $N \times M$ indicator matrix, which is 1 if the individual is heterozygous at the SNP and 0 otherwise. Errors ${\mathbf{E}}$ are assumed normally distributed. Let $\tilde{\theta }_{j}$ be the effect of SNP $j$, with $\tilde{\theta }_{j} = \left( {\tilde{a}_{j} ,\tilde{d}_{j} } \right)$. It was assumed that the distribution of $\tilde{\theta }_{j}$ is a mixture of two distributions ($F$) that differ only by a scaling factor $\varepsilon$. The form of the distribution *F* is described in detail in [18]. Conditional on a Bernoulli-distributed indicator variable $\gamma_{j}$, we have $\tilde{\theta }_{j} |\gamma_{j} \sim\gamma_{j} F + \left( {1 - \gamma_{j} } \right)\varepsilon F$. Thus, if $\gamma_{j} = 1$, the marker effect comes from the distribution that has the larger variance. The prior probability of a marker being important (i.e. $\gamma_{j} = 1$) was $pLD$. The distribution of the absolute value of additive effects was assumed to follow a folded $t$-distribution. The distribution of the dominance effect was assumed normal conditional on the absolute additive effect. The prior distribution of the dominance effects was conditional on the additive effects and was specified such that the absolute additive effects and dominance coefficients ($h = d/\left| a \right|$) are independent, which implies that the probability that a dominance effect is much larger in magnitude than the additive effect is small. As a result, presence of overdominance ($h > 1$) is a rare but non negligible event. The prior probability of the sign of the additive effects depends on the allele frequency. This probability was chosen such that it is unlikely that the genetic variance of the gene is large. This is the assumption made in BayesD, because selection has shifted allele frequencies away from values for which the contribution of a gene to the genetic variance is large [18]. This prior was not used in this study, because no selection was simulated.

For the joint posterior distribution and the Markov chain, which was generated by Gibbs sampling, see [18]. BayesD is an extension of the BayesC method of Verbyla et al. [16, 17] towards accounting for dominance. Hence, the model shown above was also applied for BayesC, but without the dominance term and assuming $\tilde{\theta }_{j} = \tilde{a}_{j}$.

We chose $\varepsilon = 0.01$ and $2.5$ degrees of freedom for the $t$-distribution of the additive effects for both BayesC and BayesD. A single MCMC chain was run for 20,000 cycles, discarding the first 10,000 as burn-in, using the R-package BayesDsamples (can be obtained from R. Wellmann). Every 100th sample, the additive and dominance (only for BayesD) effects were stored for inference purposes. The simulated variance components and expected inbreeding depression were used in the models as input parameters. Parameter $pLD$ was chosen such that the expected number of SNPs coming from the distribution with the larger variance approached the number of QTL when the size of the SNP panel approached the total number of SNPs in the genome. We used the following calculations for *pLD* for analysis of the simulated data. For $t = 1,  2,  3,  4.8$, we have a marker density of $M = 250 \times 2^{t}$, i.e. 0.5k, 1k, 2k, and 7k. We chose $pLD = 5.5 \times 0.7^{t} \times 15/M$. This results in a $pLD$ of 0.116, 0.040 and 0.014 for $t$ = 1, 2, and 3 respectively, and 0.0021 for $t$ = 4.8. This results in an average number of SNPs per QTL of $5.5\times 0.7^{t} =$ 3.85, 2.70, 1.89 and 1 for $t$ = 1, 2, 3 and 4.8 respectively. The same $pLD$ was used for both Bayes models.

### Inference of association

For inference of association, we used the posterior probability of the window association (WPPA) criterion proposed by Fernando and Garrick [5] and Fernando et al. [10], using a sliding window approach. The window size was varied between 0.25, 0.5, and 1 cM. For each of the stored estimates of the SNP effects from the MCMC chain in a window $w$, the genomic variance was computed as follows:

$$ \widehat{{\sigma_{{g_{w} }}^{2} }} = \mathop \sum \limits_{j \in w} \left[ {H_{j} \widehat{{\alpha_{J}^{2} }} + H_{j}^{2} \widehat{{d_{J}^{2} }})} \right], $$

where $H_{j} = 2p_{j} q_{j}$ is the heterozygosity of SNP $j$, $\widehat{{\alpha_{J} }} = \widehat{{a_{J} }} + \left( {q_{j} - p_{j} } \right)\widehat{{d_{J} }}$ is the estimated substitution effect of SNP $j$, and $\widehat{{a_{J} }}$ and $\widehat{{d_{J} }}$ are the corresponding estimated additive and dominance SNP effects. For BayesC, these computations were done without including estimated dominance effects, because they were not estimated in BayesC. Note that the estimated effects can be inserted into Eqs. (1) to (3) if estimated genomic breeding values, estimated genomic dominance deviations or estimated genetic values are of interest. The summation in Eqs. (1) to (3) is then over $M$ SNPs instead of over $Q$ causative mutations.

For each window, the ratio $q_{w}$ is calculated as:

$$ q_{w} = \widehat{{\sigma_{{g_{w} }}^{2} }}/E\left( {\sigma_{{g_{w} }}^{2} } \right), $$

where $$ E(\sigma_{{g_{w} }}^{2} ) = \sum\nolimits_{j \in w} {[H_{j} E(\alpha^{2} ) + H_{j}^{2} E(d^{2} )]} $$.

Expectations $E\left( {\alpha^{2} } \right)$ and $E\left( {d^{2} } \right)$ were derived under the assumption of an equal distribution of the total genetic variance across the genome, as shown in the “Appendix”. Ratios $q_{w}$ were computed for both BayesD and BayesC by using the same definition and calculation of $E\left( {\sigma_{{g_{w} }}^{2} } \right)$ (i.e. the same value was used for both BayesC and BayesD). If $q_{w} > 1$, this indicates the presence of a causative mutation within window $w$ with an effect greater than expected under the assumption of an equal distribution of the genetic variance across the genome. Hence, causative mutations with effects below this expectation are in general not detectable. The posterior distribution of $q_{w}$ was approximated by the distribution of the $q_{w}$ obtained from the stored MCMC samples of the additive and dominance effects. The WPPA was calculated by counting the number of samples for which $q_{w} > 1$ and dividing this by the total number of samples saved. The following three levels of WPPA were considered: 0.85, 0.95, and 0.99. According to [5, 10], this controls the proportion of false positives (PFP) at ≤0.15, ≤0.05, and ≤0.01, respectively.

A causal gene is mapped if at least one window within a region of 1 cM surrounding the gene shows a WPPA above the threshold. For each simulated trait, power to map a causal gene was calculated by dividing the number of mapped causative genes by the number of simulated causative genes that showed an effect greater than expected under the assumption that the genetic variance is distributed equally across the genome. Hence, very small causative genes were not counted. This guarantees that the upper bound of the power is 1, as it should be. From the definition of $q_{w}$, it becomes clear that this would not be the case if all simulated causative genes were counted. In addition, power was calculated only for causative genes that explained more than 2.5% of the simulated genetic variance, which is denoted as large-power (L-power). Mapping precision was calculated for each simulated trait as the size of the genome around a mapped causative gene with significant sliding window(s).

### Application to a real dataset

The models were also applied to a Fleckvieh dairy cattle dataset, which is described in detail by Ertl et al. [12]. In brief, the dataset included 1996 cows with yield deviations (YD) for milk production and conformation traits. The YD observations were based on test-day observations adjusted for non-genetic effects, but not for the permanent environmental effect. The cows were genotyped with the Illumina BovineHD genotyping BeadChip. After quality control, 629,028 SNPs remained in the dataset. This dataset was a subset of the data used for prediction purposes [19]. In that study, milk fat yield was chosen to compare G-BLUP and BayesD with regard to their ability to predict a cow’s YD accurately. For this trait, it is known that the *DGAT1* gene has a major effect in this population and dominance is important [12]. Therefore, this trait was also chosen in our study. The narrow sense heritability was equal to 0.47 and the dominance variance was equal to 0.18 as a proportion of phenotypic variance [12]. Parameter $pLD$ was set to 0.05 and 2.5 degrees of freedom were chosen for the $t$-distribution of the additive effects [19].

## Results

### Simulated datasets

Results of the power evaluations from the simulated datasets are in Table 1. Standard deviations are also included in this table, from which the standard errors can be calculated, if desired. Power decreased as the WPPA level increased for all simulated configurations and for both methods, as expected. L-power was substantially higher (about 0.2 across all results) than power. This indicates that it is very unlikely to find the numerous small simulated causative effects, even for low WPPA levels and with sequence data, as mimicked by the 7k dataset.

**Table 1:** Power and L-Power^a^ as a function of the SNP density per M, window size in cM, window posterior probability of association (WPPA) and method (BayesC and BayesD)

SNP density (k/M)	WS	WPPA	BayesC		BayesD	
			Power	L-power^a^	Power	L-power^a^
0.5	0.25	0.85	0.261 (0.129)	0.441 (0.199)	0.282 (0.132)	0.475 (0.205)
		0.95	0.201 (0.107)	0.347 (0.169)	0.217 (0.110)	0.362 (0.154)
		0.99	0.144 (0.102)	0.249 (0.166)	0.175 (0.101)	0.300 (0.170)
	0.5	0.85	0.267 (0.138)	0.445 (0.193)	0.280 (0.120)	0.480 (0.205)
		0.95	0.205 (0.107)	0.349 (0.174)	0.230 (0.103)	0.388 (0.158)
		0.99	0.151 (0.094)	0.260 (0.151)	0.192 (0.099)	0.336 (0.182)
	1	0.85	0.279 (0.142)	0.467 (0.204)	0.298 (0.135)	0.500 (0.193)
		0.95	0.214 (0.114)	0.352 (0.156)	0.241 (0.102)	0.410 (0.161)
		0.99	0.167 (0.092)	0.285 (0.146)	0.199 (0.095)	0.343 (0.165)
1	0.25	0.85	0.265 (0.119)	0.452 (0.184)	0.290 (0.112)	0.500 (0.186)
		0.95	0.210 (0.114)	0.365 (0.194)	0.241 (0.101)	0.415 (0.173)
		0.99	0.193 (0.112)	0.337 (0.196)	0.180 (0.091)	0.315 (0.157)
	0.5	0.85	0.291 (0.121)	0.495 (0.190)	0.321 (0.123)	0.551 (0.204)
		0.95	0.228 (0.116)	0.394 (0.199)	0.265 (0.108)	0.454 (0.180)
		0.99	0.192 (0.106)	0.334 (0.174)	0.198 (0.088)	0.344 (0.151)
	1	0.85	0.316 (0.128)	0.538 (0.208)	0.341 (0.139)	0.580 (0.213)
		0.95	0.259 (0.116)	0.438 (0.171)	0.291 (0.125)	0.497 (0.204)
		0.99	0.216 (0.119)	0.369 (0.183)	0.227 (0.096)	0.388 (0.145)
2	0.25	0.85	0.285 (0.126)	0.481 (0.176)	0.299 (0.126)	0.501 (0.169)
		0.95	0.247 (0.115)	0.423 (0.172)	0.245 (0.107)	0.419 (0.155)
		0.99	0.216 (0.109)	0.367 (0.159)	0.214 (0.106)	0.325 (0.169)
	0.5	0.85	0.332 (0.118)	0.561 (0.169)	0.343 (0.129)	0.571 (0.168)
		0.95	0.276 (0.118)	0.469 (0.182)	0.284 (0.115)	0.476 (0.152)
		0.99	0.234 (0.119)	0.395 (0.172)	0.221 (0.114)	0.376 (0.176)
	1	0.85	0.348 (0.130)	0.586 (0.181)	0.371 (0.141)	0.618 (0.182)
		0.95	0.312 (0.132)	0.522 (0.179)	0.318 (0.117)	0.532 (0.147)
		0.99	0.245 (0.123)	0.406 (0.178)	0.248 (0.121)	0.420 (0.172)
7	0.25	0.85	0.307 (0.120)	0.518 (0.185)	0.301 (0.093)	0.505 (0.164)
		0.95	0.273 (0.097)	0.467 (0.156)	0.263 (0.092)	0.449 (0.166)
		0.99	0.259 (0.090)	0.435 (0.146)	0.256 (0.075)	0.393 (0.146)
	0.5	0.85	0.356 (0.107)	0.602 (0.172)	0.369 (0.099)	0.615 (0.168)
		0.95	0.309 (0.102)	0.518 (0.161)	0.312 (0.093)	0.531 (0.142)
		0.99	0.270 (0.089)	0.459 (0.165)	0.273 (0.089)	0.460 (0.135)
	1	0.85	0.378 (0.118)	0.636 (0.152)	0.394 (0.106)	0.657 (0.137)
		0.95	0.329 (0.116)	0.551 (0.156)	0.339 (0.103)	0.567 (0.152)
		0.99	0.281 (0.109)	0.465 (0.151)	0.279 (0.095)	0.472 (0.155)

Standard deviations are in parenthesis

^a^L-Power denotes the power to detect a causal gene that explains more than 2.5% of the simulated genetic variance

*WS* window size

Power increased with SNP density. For example, for BayesC, a WPPA of 0.95 and a window size of 0.5, L-power was equal to 0.35, 0.39, 0.47, and 0.52 for SNP densities of 0.5k, 1k, 2k, and 7k, respectively. The same pattern was observed for BayesD.

Power also increased as window size increased. For example, L-power was equal to 0.42, 0.47, and 0.52 for window sizes of 0.25, 0.5 and 1 cM respectively (2k SNP density, 0.95 WPPA and BayesC). This pattern held true for all genetic configurations and for both methods.

Application of BayesD improved the power compared to BayesC for almost all genetic configurations. For example, for a WPPA of 0.95 and a window size of 1 cM, L-power of BayesC was equal to 0.35, 0.44, 0.52, and 0.55 and that of BayesD was equal to 0.41, 0.5, 0.53, and 0.57 for SNP densities of 0.5k, 1k, 2k and 7k, respectively. In general, it appeared that the superiority of BayesD over BayesC declined as SNP density increased. For a 7k SNP density, and a window size of 0.25, BayesC resulted in slightly greater power than BayesD.

Results of the evaluation of mapping precision are in Table 2. Because mapping precision was not affected by the WPPA level, except for some random fluctuations (not shown), they are reported as means across all WPPA levels. By definition, the lower bound of precision was the window size and, thus, window size affected the precision. For example, for BayesC (2k SNP density and 0.95 WPPA), a precision of 0.51, 0.93, and 1.75 cM was obtained for window sizes of 0.25, 0.5, and 1 cM, respectively. Precision was improved, although only slightly, with an increase in SNP density. For example, for BayesC and a window size of 0.5 cM, precisions of 1.1, 0.94, 0.93, and 0.91 cM were obtained for SNP densities of 0.5k, 1k, 2k, and 7k, respectively. BayesD improved mapping precision only slightly and not in all cases. In general, precision was slightly better for BayesD for smaller window sizes. However, with a window size of 1 cM, precision was slightly better for BayesC. A remarkable outcome is that even with sequence data, the precision was substantially higher than its lower bound. This held true for both methods.

**Table 2:** Precision as a function of SNP density per M, window size in cM, and method (BayesC and BayesD)

SNP density (k/M)	WS	BayesC	BayesD
0.5	0.25	0.653 (0.223)	0.639 (0.219)
	0.5	1.050 (0.275)	1.047 (0.245)
	1	1.779 (0.283)	1.796 (0.259)
1	0.25	0.525 (0.130)	0.517 (0.095)
	0.5	0.943 (0.138)	0.943 (0.152)
	1	1.720 (0.215)	1.744 (0.182)
2	0.25	0.506 (0.138)	0.467 (0.085)
	0.5	0.930 (0.125)	0.909 (0.121)
	1	1.746 (0.184)	1.728 (0.157)
7	0.25	0.468 (0.042)	0.449 (0.046)
	0.5	0.912 (0.074)	0.906 (0.075)
	1	1.798 (0.120))	1.802 (0.114)

Standard deviations are in parenthesis

*WS* window size

### Real dataset

**Fig. 2:**
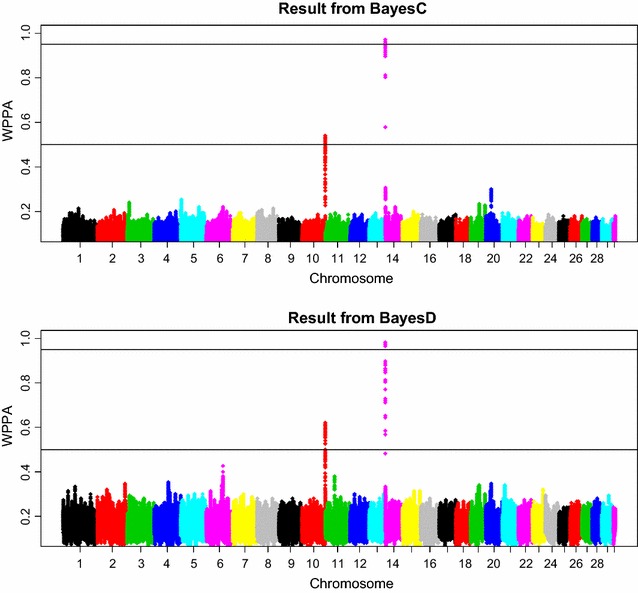
Plot of window posterior probabilities of association (WPPA) obtained by BayesC (*top*) and BayesD (*bottom*), from the real data analyses

Plots of WPPA along the chromosomes are in Fig. 2 for a window size of 0.5 cM. For the two other window sizes (0.25 and 1 cM), similar plots were obtained, except that the peaks were somewhat more (less) pronounced with a window size of 1 cM (0.25 cM) (not shown). Both methods produced a clear signal on BTA14 at the chromosomal position where *DGAT1* is located and a clear signal on chromosome 10, although this was below the threshold levels used in this study. No other WPPA was above the threshold levels used in this study, which indicates that either no other genes with a larger effect are segregating in this population, or the sample size of the data set is too small, or both.

**Fig. 3:**
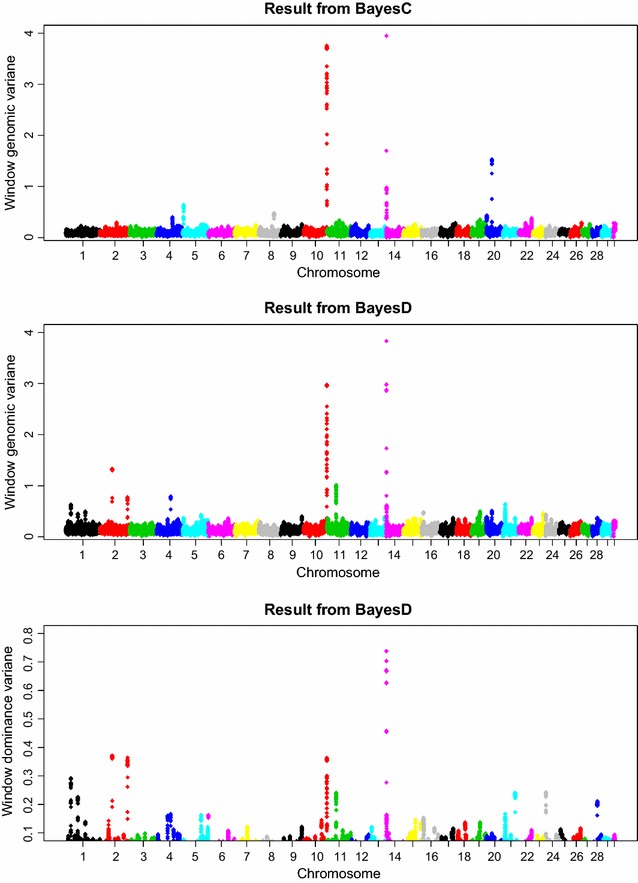
Estimates of window genomic variances based on the real data analyses. The *top* and *middle panels* show the within-window estimates of genomic variances obtained by BayesC and BayesD, respectively. The *bottom panel* shows the within-window dominance variance obtained by BayesD. The window variances were multiplied by 1000. The window size was 0.5 cM

Closer inspection of the WPPA plots revealed some differences between the two methods. BayesD produced several extra signals, although far below the threshold levels used in this study. Examples are on chromosomes 6, 11, and 21. In addition, the average WPPA across the genome was slightly higher for BayesD than for BayesC. In Fig. 3, estimates of the window additive genetic variance of the window dominance variance are provided. From this figure it seems that not only the additive effects are spread across the genome, but also the dominance effects.

## Discussion

### Simulation protocol

Multi-marker Bayesian methods for association analyses were compared using simulated and real data. The simulation protocol regarding genetic architecture followed our current understanding of quantitative traits with regard to number of segregating causal mutations and their additive and dominance effects. In a recent study [15], we conducted an in-depth theoretical analysis of the contribution of dominance to the variation of quantitative traits. One aim of that analysis was to develop a simulation protocol that models dominance gene effects that result in realistic genetic variance components and to validate this protocol with a sensitivity analysis. The current simulation protocol follows the recommendations given in that study.

Only one chromosome was simulated because the MCMC-based analyses were computationally demanding and replicated simulations were performed. It is possible that the power that would result from the simulation of multiple chromosomes would be somewhat reduced, but this would not alter the general findings of our study. However, the results obtained by using real data revealed that it is possible to also apply these methods in the case of genomes that consist of numerous chromosomes, and for which 630k SNPs are available. Due to the stochastic nature of the effects during the simulation of the traits, the dominance variance calculated as the proportion of the phenotypic variance ranged from 0.01 to 0.29, with a median of 0.08. These values are consistent with those reported by Bolormaa et al. [14], i.e. proportions from 0 to 0.42, with a median of 0.05, and indeed also with the estimates obtained from the dairy cow dataset that was used here. For some simulated traits, dominance was not important at all, which is also the case for real quantitative traits. We favoured this stochastic approach of simulating dominance variance proportions instead of choosing fixed proportions, because this generalised the results obtained for typical quantitative traits instead of producing results that are valid only for a defined dominance variance proportion.

### BayesC versus BayesD

The results showed that method BayesD increased power to map QTL compared to BayesC in almost all analyses because it uses dominance variance as an additional source of genetic variance. This increased power can also be deduced from the results of the analyses of the real dairy cow dataset (Fig. 2), where BayesD produced several additional signals that were not found in the BayesC results.

**Fig. 4:**
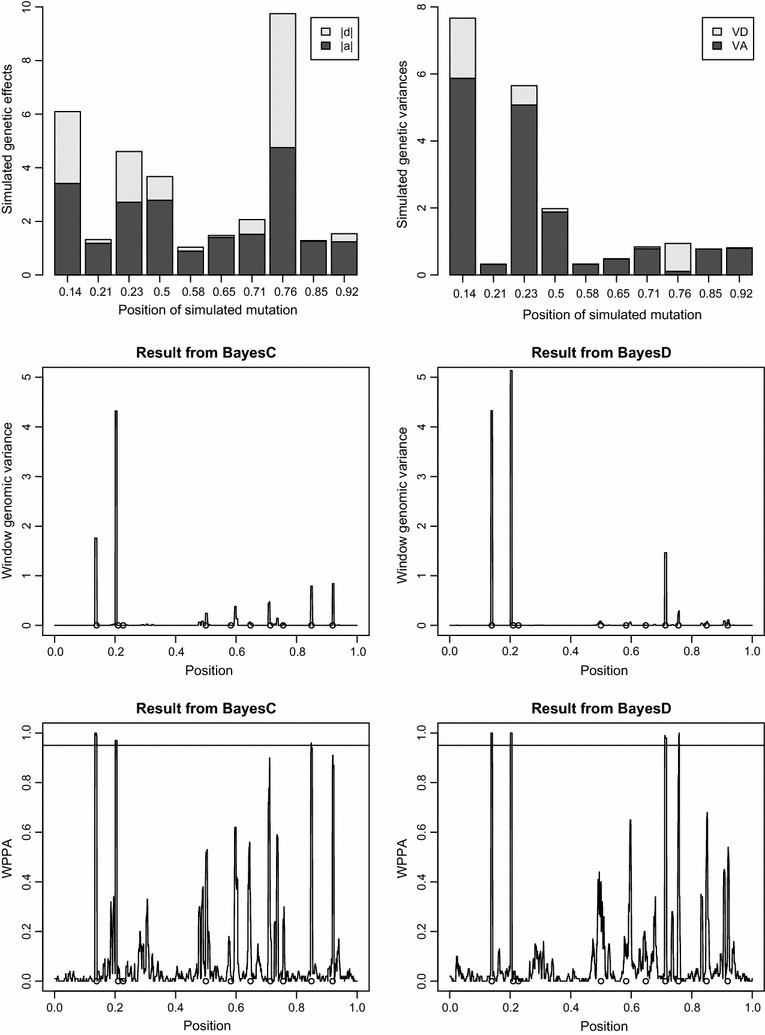
Simulated gene effects and BayesC and BayesD results for a single simulated trait. The *top left panel* shows the simulated additive and dominance effects of the 10 causative mutations with a non-negligible effect for a randomly chosen trait for which dominance was important. The *top right panel* shows the genetic variances of these simulated causative mutations. The *two panels in the middle* show the within-window genomic variances obtained by BayesC (*left*) and BayesD (*right*). The window posterior probability of association (WPPA) obtained from both methods are shown at the *bottom*. The positions of the 10 causative mutations are indicated by a *circle*

Figure 4 shows the genetic effects and genetic variances of simulated causal mutations for a randomly chosen simulated trait for which dominance was important. Plots of the estimated window genomic variance across the chromosome and of the WPPA are also in Fig. 4. The two large causal mutations at positions 0.14 and 0.23 were detected with both methods (WPPA > 0.95), although both showed a relatively large dominance variance and the estimate of genomic variance was substantially larger for BayesD than BayesC. For the causal mutations with a smaller effect size at positions 0.71 and 0.76, dominance was important and these mutations were only significant with BayesD (WPPA > 0.95). For the mutation with a moderate effect size at position 0.85, dominance was not important and this mutation was only detected with BayesC (WPPA > 0.95). These examples illustrate the following general findings: mutations with a large effect are likely to be detected by both methods, mutations with a moderate effect and with dominance effects are more likely to be detected by BayesD, and those without dominance by BayesC. Mutations with a small effect are not detected by either method. Hence, although the power of BayesD is generally higher than that of BayesC, the application of both models will likely improve overall power.

Since dominance is an interaction effect of the two alleles at a locus, their effects are captured in the association analysis by matched haplotype pairs, i.e. diplotypes. Diplotypes show a faster breakup around a focal point in the genome compared to haplotypes. Hence, BayesD was expected to improve the mapping precision as well, but the observed improvement was only small and was more pronounced for smaller window sizes.

For a high SNP density and a small window size, BayesC outperformed BayesD with regard to power. This intuitively unexpected result can be explained by the fact that, in BayesC one effect is estimated per SNP, whereas in BayesD two effects are estimated per SNP. Thus, with BayesD, the effect of a causative mutation may be spread over more very closely-linked SNPs than for BayesC, and some SNPs may even be outside the window, if the window size is small.

For BayesC, only the additive genetic variance and the residual variance need to be known, which can usually be estimated from the data with high precision. In BayesD, dominance variance and inbreeding depression also need to be known. With the use of genomic data it might be possible for most traits in most populations to estimate them precisely, at least for the dominance variance [12, 13]. Common to both methods is the specification of the number of degrees of the $t$-distribution of the SNP effects. We chose relatively small degrees in order to obtain a heavy-tailed distribution and thus clearer association signals. In addition, parameter $pLD$, i.e. the prior probability that an SNP effect comes from the distribution with the large variance, needs to be specified. In this study, for the simulated datasets, this was specified under the assumption that the expected number of required SNPs approaches the number of causative mutations when the size of the SNP panel approaches the total number of SNPs. Alternatively, the parameter could have been estimated from the data [26] but the current version of the MCMC algorithm does not include this option [18], or by using grid searches. In the real dataset, the input parameters were taken from an earlier study [19], in which these parameters were varied across a specified range of values. The parameters that need to be specified for the Bayesian methods are not needed in single-marker GWAS implemented in linear mixed models. This makes the application of these models more convenient and is probably one reason why these methods are more widely used.

### False positive results

Our simulation study was not designed to determine whether the WPPA controls the proportion of false positives at the desired level. This hampered the comparison of WPPA between BayesC and BayesD in a formal way. Instead, we searched for elevated WPPA (at the lowest threshold level of 0.85 and even lower) in chromosomal regions of about 10 cM that had no simulated causal mutation. In very exceptional cases, an elevated WPPA was observed in these regions without any differences between BayesC and BayesD. Hence, it appears that WPPA controls the proportion of false positives in the simulation study at a low level for both methods. This might be due to the small number of degrees of freedom that was used for the $t$-distribution of the SNP effects, which allows large true effects to be detected, but small and spurious effects are regressed back to zero and, hence, are not detected. A larger number of degrees of freedom would probably have resulted in greater power but also in a larger number of false positives. From this, it became also obvious that the frequentist properties (power, false positive rate) of these methods remains somewhat unclear, because the WPPA criterion as implemented in this study appeared to be a poor guide for the false positive rate.

### Window approach

Instead of drawing inferences from SNPs in windows, the posterior probability of the effect of a specific SNP being drawn from the distribution with the larger variance could have been used for inference purposes. However, in the study of Sahana et al. [8], this resulted in reduced power, because the gene effects may be distributed over several consecutive SNPs and individual SNPs thus have a reduced power. Thus, the window approach is a logical consequence of applying multi-marker Bayesian methods for GWAS with dense SNP panels. Window size affected the power and precision in opposite directions, i.e. power increased with window but precision was lower for larger window sizes. Hence, there is a trade-off between these two criteria of success. An obvious solution would be to start with larger windows, e.g. of 1 cM, to find significant chromosomal regions that are associated with a trait and subsequently reduce window size to fine-map the causal mutations. With full sequence data, it can be assumed that the causal mutation is among the genotypes. Obviously, pinpointing the causal mutation within a fine-mapped region is not possible with a window approach. In this case, it might be beneficial to use posterior probabilities for individual SNPs [7], as well as other methods [27, 28]. Although complete sequence information for each individual was available in the 7k dataset, we did not attempt to detect the causal mutation within a fine-mapped region.

A sliding-window-based approach was used by moving the window boundaries one SNP forward along the chromosome. This resulted in as many windows as SNPs included in the analysis, and also in the same number of WPPA, which are highly correlated for consecutive windows. Alternatively, the chromosome could have been divided into non-overlapping windows. This would reduce the number of windows but introduces the problem of arbitrarily setting the window boundaries. It is possible that such boundaries will break a chromosomal region that harbours a causative mutation into two or more distinct windows, which would reduce the power to map the mutation. The choice of windows deserves additional investigations that take the LD structure along the genome into account [29]. Another option would be to fit haplotypes [30]. This would also extend the inferences beyond a single SNP but the SNPs would be in strong LD.

Inferences were drawn by using the WPPA criterion and using estimates of genomic variance within windows. If inference on the importance of dominance is of interest, the same criterion can be used by using estimates of dominance variance within windows. A straightforward strategy would be to map causative genes using estimates of within-window genomic variance, as done in this study, and then test the importance of dominance by using the within-window estimate of dominance variance.

### Large datasets are needed

Given that the scaling argument of [21] also holds at least approximately, for GWAS, large-scale populations were simulated when scaling towards a bovine genome of 30 M and an Ne of 100 or 1000. Results show that, even with these large datasets, power to find causative genes that explain more than 2.5% of the genetic variance (L-power in Table 1) is above 0.5 only in a few configurations. Hence, our findings show that large datasets are needed if also moderate and eventually small effect associations are to be detected. Results of the real data analyses support this as well. BayesD needs genotypes and trait measurements from the same individual. In cattle, this means that cows need to be genotyped and this is becoming part of the routine genotyping strategy in many dairy cattle breeding organisations. Hence, it can be expected that such large-scale datasets that include genotyped cows will soon be available.

## Conclusions

The application of BayesD for GWAS on simulated quantitative traits with realistic dominance effects resulted in increased power compared to BayesC. The increase in power was between −2 and 8% for causative genes that explain more than 2.5% of the genetic variance. This trend in increased power was also observed in the results from the real data analyses. In addition, by examining the within-window estimates of genomic dominance variance, BayesD allows for inference about the magnitude of dominance effects at significant associations, which remains hidden in BayesC analyses. Mapping precision was, however, not substantially improved by BayesD. If the aim is to map mutations with medium and small effects, large datasets that include several thousands of individuals are needed.

## Appendix

In this appendix, we show how to derive expectations $E\left( {\alpha^{2} } \right)$ and $E\left( {d^{2} } \right)$ under the assumption of an equal distribution of the additive genetic variance ($V_{A}$) and of dominance variance ($V_{D}$) across all SNPs $M$. Under this assumption, the expected additive genetic variance of a marker $m$ can be denoted by $E\left( {V_{{A_{m} }} } \right) = \frac{{V_{A} }}{M}$ and the expected dominance variance by $E\left( {V_{{D_{m} }} } \right) = \frac{{V_{D} }}{M}$. Furthermore, $E\left( {V_{{A_{m} }} } \right) = E\left( {2pq} \right) \times E\left( {\alpha^{2} } \right)$ and $E\left( {V_{{D_{m} }} } \right) = E(\left( {2pq} \right)^{2} ) \times E\left( {d^{2} } \right)$. The expectation of $2pq$ can be approximated by the mean of the heterozygosity across all SNPs and is denoted by $\bar{H}.$ Similarly, the expectation of $\left( {2pq} \right)^{2}$ can be approximated by calculating the squared heterozygosity of each SNP and subsequently by taking the mean of this value across all SNPs. This is denoted as $\overline{{H^{2} }}$. Thus, $E\left( {\alpha^{2} } \right) = \frac{{V_{A} }}{{M\overline{H} }}$ and $E\left( {d^{2} } \right) = \frac{{V_{D} }}{{M\overline{{H^{2} }} }}$, which was used for the calculation of $q_{w}$ in the main text.
